# Green space quantity and exposure in relation to the risk of immune-mediated diseases: a scoping review

**DOI:** 10.1186/s12889-024-20655-x

**Published:** 2024-12-02

**Authors:** Polina Galitskaya, Anna Luukkonen, Marja I. Roslund, Miia Mänttäri, Anja Yli-Viikari, Liisa Tyrväinen, Aki Sinkkonen, Olli Laitinen

**Affiliations:** 1 Research Institute for Environmental Sciences (RIES), Parede, Portugal; 2 Natural Resources Institute Finland, Helsinki and Turku, Turku, Finland; 3 Tampere University, Tampere, Finland

**Keywords:** Immune-mediated and inflammatory diseases, Green area, Green space, Green cover, Biodiversity hypothesis, NDVI, Land use and land cover

## Abstract

The growing global incidence of immune-mediated and inflammatory diseases (IMIDs) is worrisome, with evidence suggesting that environmental factors, notably urbanization and the reduction of green spaces, may act as potential instigators. However, conflicting findings in studies necessitate a closer examination of recent research (January 2020 – February 2024) to elucidate the factors contributing to these inconsistencies. This review explores study protocols to avoid erroneously endorsing the null hypothesis of no association between green space coverage and IMID risks. A literature search adhering to PRISMA-ScR guidelines yielded 46 relevant papers from Google Scolar and Pub Meb. The studies varied in design, with 17 being longitudinal, 24 cross-sectional, and five focusing on longitudinal parent-offspring connections. Geographic scope differed, with 21 multi-location and 25 single-location studies. Participant numbers ranged from 144 to 982,131 across diverse demographics. Additionally, some studies examined disease frequencies in large groups (several million people) residing in specific regions. Green space metrics encompassed NDVI, land cover data, plant biodiversity, and novel indexes, measured within 7.5–5000 m diameter buffers around residences or schools. The review advises against making definitive statements regarding the relationship between urban green spaces and the prevalence of IMIDs. It suggests that inconsistencies in study results may stem from variations in study designs and methodologies, as well as the complex, interacting mechanisms through which green spaces affect immune health. Future research recommendations include larger cohorts, early-life exposure data, and testing specific hypotheses related to vegetation types and participants’ genetic predispositions.

**Supplementary Information:**

The online version contains supplementary material available at 10.1186/s12889-024-20655-x.

## Introduction

 Presently, approximately half of the global population resides in urban areas, and by 2050, this proportion is projected to increase to 65% [1]. The urban lifestyle raises serious concerns due to its association with processed food, higher levels of air and water pollution, reduced physical activity, and decreased exposure to natural environments, particularly those covered with vegetation (such as green areas and spaces) [2–5]. Low exposure to natural environments is considered a significant risk to human health, as nature is associated with mitigating stress, reducing environmental hazards, enhancing physical activity and social interaction, and providing exposure to diverse environmental microbial communities [2–5].

Immune-mediated and other inflammatory diseases (IMIDs) encompass a broad group of multifactorial conditions with diverse symptoms, including rheumatoid arthritis, psoriasis, atopic dermatitis, type 1 diabetes, asthma, allergic rhinitis, and certain neurological diseases. The unifying feature of these diseases is the dysregulation of the immune system [6, 7]. IMIDs can be broadly categorized into two major classes. The first class comprises autoimmune diseases, in which the immune system mistakenly attacks the body’s own cells and organs, as seen in type 1 diabetes and celiac disease. The second class involves erroneous activity against non-harmful environmental factors, such as pollen and animal dander, with atopic disorders being typical examples of this category. Additionally, there are many other inflammatory diseases where the immune system plays a role, but which cannot be clearly categorized into either of these classes. The causes of immune-mediated diseases are varied, but they essentially fall into two broad groups: genetic and environmental factors. Environmental factors can either trigger or accelerate the clinical manifestation of these diseases [8]. On the other hand, environmental factors can also protect from the diseases [9]. Given the significant rise in IMIDs in recent years, it is posited that rapid shifts in environmental factors, as opposed to extremely slow alterations in genetics, play a pivotal role in this increase [10]. Interestingly, since environmental factors can induce epigenetic changes, they have the potential to affect subsequent generations, positioning them at the intersection of these influences. Considering the population migration to urban areas, which are characterized by lower levels of natural environments, the lack of exposure to nature is believed to be one of the key factors driving the increase of the IMIDs.

Asthma, the most common childhood respiratory disease, is estimated to affect over 400 million people worldwide by 2025 [11, 12]. Recently, over 250 thousands people die yearly from asthma or its consequences [13]. Allergic rhinitis is the most common allergic disease worldwide. Despite its seemingly modest effect on health, it significantly impacts quality of life and work productivity, leading to substantial economic losses and increased healthcare expenditures [14, 15]. Type 1 diabetes is one of the most widespread metabolic disorders in children. According to recent data, this disorder has a genetic predisposition that, in 10% of cases, progresses to the disease. The main potential factors influencing this progression are perinatal conditions, infections, and the environment [16, 17]. Genetically predisposed acute lymphoblastic leukemia is the most common cancer type in children. It progresses to the acute phase in 1% of genetically predisposed children, and this progression is triggered by a dysregulated immune response to viral and bacterial infections [18]. Interestingly, the maturation of the immune system, facilitated by exposure to diverse microbial communities, is demonstrated to significantly reduce this dysregulation [19].

Recently, numerous studies have been published linking green spaces to various aspects of human health. Beneficial associations have been demonstrated between the quality and quantity of green spaces and outcomes such as mortality, mental health, birth weight, body mass index, and sleep quality [2, 20]. Fewer studies have focused on the associations between green spaces and IMIDs. One of the first reviews examining the relationship between urban green spaces and allergic respiratory diseases was published in 2017 by Lambert with co-authors [21]. This review conducted a meta-analysis of data from 11 articles concerning asthma and allergic rhinitis. The authors concluded that the results were inconsistent, making it impossible to accurately assess the association between green space coverage and the level of allergic respiratory diseases. In 2018, Lambert and co-authors published another review on allergy and atopic sensitization in children, analyzing five articles that together covered eleven cohorts. A lower risk associated with the vicinity of vegetation was reported in four of these studies, a higher risk in two, and no change in risk in five [22]. Furthermore, Hartley et al. published a review in 2020 on the effects of green spaces on childhood asthma, analyzing seven primary research articles. Six of these articles did not reveal any association, while one reported a protective association between green areas and childhood asthma [13]. Ferrante with co-authors (2020) published a review on effects of residential urban green spaces on allergic respiratory diseases in youth [23]. It included 14 publications published up to December 2018. Four of them showed an increased risk of asthma in areas with higher relative coverage of green space, three reported the opposite effect, and the remaining publications did not reveal significant effects. The authors concluded that inconsistencies in the results, due to differences in experimental designs, methods of estimating exposure, geographic regions, and the sizes of groups used by researchers, hamper the comparability of research outcomes. Mueller et al. published an exploratory review in 2022 on urban greenspace associations with respiratory health that included 108 primary publications [24]. It only partly included information about IMIDs, in particular asthma. Over half of the publications related to asthma indicated either a significant or an insignificant reduction in asthma risks associated with greenspaces. Wu and co-authors performed a meta-analysis of 21 studies in 2022, investigating the relationship between green space exposure and the incidence of asthma and allergic rhinitis. Their findings indicated no significant effects of green space exposure on either disease. The authors suggest that variations in disease diagnosis, methods of measuring green spaces, and the confounding factors accounted for in the studies may influence these outcomes [25]. A similar relationship, but regarding children, was the focus of a study by Liu and co-authors in 2023 [26]. Their systematic review and meta-analysis included 23 original articles, and the results were contradictory. Depending on the area used by researchers around the residence of the subjects, the association between asthma and greenness was either negative or nonexistent. Allergic rhinitis, on the other hand, manifested differently in children of different ages. In the systematic review and meta-analysis by Tang et al. (2023) that included 35 publications, the impact of green spaces on chronic respiratory health issues including asthma was examined [27]. This work found a link between increased exposure to green spaces and a lower risk of certain chronic respiratory conditions, particularly in the case of asthma. Furthermore, the findings suggest that variations in age and the size of surrounding green areas may influence the relationship between green space exposure and respiratory health. The latest review and meta-analysis from 2024, published by Squillacioti and co-authors, focuses on the exposure to greenness and its effects on respiratory outcomes among youth in Europe [28]. It encompasses 24 studies and posits that no significant association was found between the greenness and an increased prevalence of asthma.

Despite numerous primary research studies and reviews exploring the connections between green spaces and IMIDs, there is currently no consensus on whether these spaces provide protective or risk-increasing associations. Both original studies and reviews exhibit a variety of focuses in terms of the type of immune disease investigated, as well as differing perspectives on the method for assessing green spaces. The most common metrics used are NDVI (Normalized Difference Vegetation Index), land cover, and tree canopy, but more specific methods are also employed, taking into account factors such as the season of exposure, its duration, the diversity of plant species, and others [24, 26]. Efforts are being made to compare the mentioned metrics and to identify the assessment methods of green spaces that are most relevant for detecting the relationship between green areas and immune-mediated diseases [29, 30]. Additionally, the choice of the area impacting the subjects under study is crucial. Researchers may select a buffer zone with a radius of 100–500 m up to 5–10 km, which can significantly influence the findings [31, 32]. The current review compiles the most recent studies conducted between January 2020 and February 2024, focusing on research methodologies that either reveal or fail to identify associations between green spaces and the risk of IMIDs. Instead of delving into the potential effects of green space exposure on this risk or the mechanisms behind such effects, this review concentrates on methodological aspects. In this review, we analyzed primary studies (case studies, research articles), took into account secondary studies (reviews and meta-analyses), and considered tertiary studies (umbrella reviews).

The main distinction of our review from previous ones is our focused attention on the methods used to evaluate green space. We hypothesized that certain methods might be more effective than others in detecting associations between IMIDs and the relative coverage of green space. Additionally, we assumed that the quality (type) of green space is crucial in determining these potential associations. One of our primary goals was to outline standards for future research or, at the very least, to assess whether and how such standards could be developed.

## Methods

The search has been done through two international electronical databases – Google Schoolar and Pub Meb - for articles published between January 2020 and February 2024. The search strategy corresponded to PRISMA guidelines for scoping reviews (PRISMA-ScR) [33]. It included combination of terms related to vegetation (greenness, green space, green area, land cover, NDVI, vegetation) and health issues (health, diseases, immune mediated disease, asthma, allergy, cardiometabolic, atopy and diabetes). Search results were exported, followed by a process of deduplication. After removing duplicates, two reviewers examined the titles and abstracts, and then conducted a full-text assessment. In instances of disagreement, a third reviewer was consulted for a final decision. The review process did not involve blinding to the journal title, authors, or affiliated institutions. A study qualified for inclusion in the review if it met the following criteria: (i) it was published in English; (ii) for observational studies, it contained at least one measurement of exposure to or proximity to green space; (iii) for intervention studies, it featured a comparison involving green area settings; (iv) it offered data on the connection between characteristics of green spaces and the risk of asthma, wheezing, allergic sensitization, rheumatoid arthritis, psoriasis, atopic dermatitis, type 1 diabetes, allergic rhinitis, or other immune-mediated inflammatory diseases (IMIDs); (v) it involved human subjects exclusively. Review articles and meta-analyses were studied for comparison but not included into analysis.

**Fig. 1:**
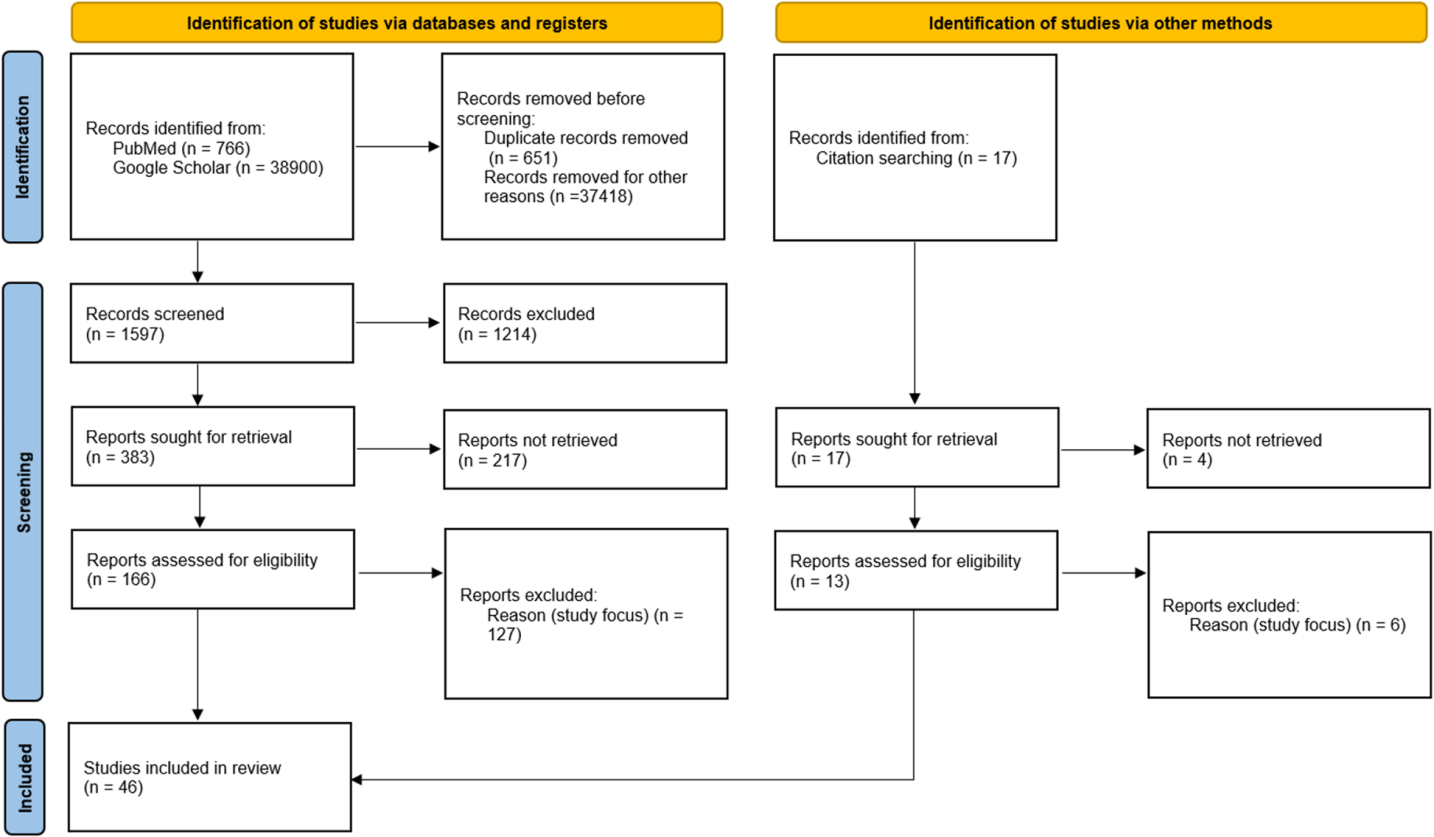
The studies selection process

References within selected studies and relevant review papers discovered in the search were examined for further studies that met the eligibility criteria. Additionally, any other references familiar to the research team, which fulfilled the eligibility requirements but were not found through the initial search strategy, were also incorporated. The process of data search is presented on Fig. 1.

From the selected articles, the following information was extracted: authors and year of publication, method of green spaces measurement, terms used to identify green spaces and health issues, place where research was conducted, time and duration of the research, immune mediated disease(s) that were analyzed, information about study participants (age, cohort, group size), the parameters that were measured and the way how they were measured, measure conclusions about association between green spaces exposure and IMID risks, potential reason for the association observed.

The evaluation of the included studies’ quality was performed using the QualSyst tool [34]. Two reviewers independently appraised the studies based on the criteria set for quantitative research, with a minimum acceptance threshold of 55% for a study’s inclusion in the review.

## Results

### General characteristics of the original research studies

46 papers met the search criteria and were included in this review [14, 16, 20, 29–32, 35–73] (Table 1, Supplement Table 1).

**Table 1:** The studies included in the review

First author and year	Title	IMID analyzed	Country	Study participants
Aerts et al., 2020 [31]	Residential green space and medication sales for childhood asthma: A longitudinal ecological study in Belgium	asthma	Belgium	1872 children characterized by sex and age group 6–12 and 13–18 years
Badpa et al., 2022 [16]	Association of long-term environmental exposures in pregnancy and early life with islet autoimmunity development in children in Bavaria, Germany	type 1 diabetes	Bavaria, Germany	85,251 children
Buchholz et al., 2023 [35]	Natural Green Spaces, Sensitization to Allergens, and the Role of Gut Microbiota during Infancy	asthma, atopy	Edmonton, Canada	1 year and 3 years of age in a cohort of 699 infants from CHILD cohort (Canada), gut microbiota was investigated at the age of 4 months
Chen et al., 2024 [36]	Greenness and its composition and configuration in association with allergic rhinitis in preschool children	allergic rhinitis	mainland China	36,867 preschool children
Cilluffo et al., 2022 [37]	Association between Asthma Control and Exposure to Greenness and Other Outdoor and Indoor Environmental Factors: A Longitudinal Study on a Cohort of Asthmatic Children	asthma	Palermo, Italy	179 asthmatic children (5–16 years)
De Roos et al., 2022 [29]	Does Living near Trees and Other Vegetation Affect the Contemporaneous Odds of Asthma Exacerbation among Asthma Patients?	asthma Pediatric	City of Philadelphia, USA	children, 17,639 exacerbation case events and 34,681 controls
Dong et al., 2021 [38]	Association between Green Space Structure and the Prevalence of Asthma: A Case Study of Toronto	asthma	Toronto (Canada)	total population of Toronto, children (0–19) and adults were analyzed separately
Donovan et al., 2021, 2021 [39]	An empirical test of the biodiversity hypothesis: Exposure to plant diversity is associated with a reduced risk of childhood acute lymphoblastic leukemia	childhood acute lymphoblastic leukemia	New Zealand	all children born in New Zealand from 1998 to 2013 (*n* = 899126; 264 ALL cases), which were followed from birth to age five
Donovan et al., 2021, 2021 [40]	The natural environment, plant diversity, and adult asthma: A retrospective observational study using the CDC’s 500 Cities Project Data	asthma	US, 500 largest cities	large national study, 500 largest cities in US
Dzhambov et al., 2021 [32]	Allergic symptoms in association with naturalness, greenness, and greyness: A cross-sectional study in schoolchildren in the Alps	asthma, allergic rhinitis, eczema	alpine valleys in Austria and Italy	1251 schoolchildren (8–12 years old)
Hartley et al., 2022 [41]	Residential greenness, asthma, and lung function among children at high risk of allergic sensitization: a prospective cohort study	asthma and lung function	Cincinnati, USA	617 children followed from birth to age 7
Hu et al., 2023 [42]	Residential greenspace and childhood asthma: An intra-city study	asthma	Shanghai, China	16,605 children aged 3–12 years
Hu et al., 2023 [43]	Higher greenspace exposure is associated with a decreased risk of childhood asthma in Shanghai - A megacity in China	asthma	Shanghai, China	16,605
Paciência et al., 2023 [44]	Varying effects of greenness in the spring and summer on the development of allergic rhinitis up to 27 years of age: The Espoo Cohort Study	allergic rhinitis	Espoo, Finland	children delivered between 1 January 1984 and 31 March 1990
Kim and Ahn, 2021 [30]	The Contribution of Neighborhood Tree and Greenspace to Asthma Emergency Room Visits: An Application of Advanced Spatial Data in Los Angeles County	asthma	Los Angeles County, USA	emergency room visits per 10,000 people across 2301 Los Angeles County census tracts
Kuiper et al., 2021 [45]	Lifelong exposure to air pollution and greenness in relation to asthma, rhinitis and lung function in adulthood	asthma	Norway, Sweden	3428 participants born after 1975 (mean age 28)
Kuiper et al., 2020 [46]	Associations of Preconception Exposure to Air Pollution and Greenness with Offspring Asthma and Hay Fever	asthma and hey fever	Respiratory Health in Northern Europe, Spain and Australia (RHINESSA): Bergen (Norway); and Umea, Uppsala, and Gothenburg (Sweden)	1106 parents born after 1975 with 1949 offspring (mean age 35 and 6)
Lee et al., 2020 [14]	Linkage between residential green spaces and allergic rhinitis among Asian children (case study: Taiwan)	allergic rhinitis	Taiwan	11,281 children (the observation strarted at the age of 4 and continiued till the age of 12)
Lee et al., 2022 [47]	Tree canopy, pediatric asthma, and social vulnerability: An ecological study in Connecticut	asthma	Connecticut, USA	Connecticut population (3605944)
Li et al., 2023 [48]	Environmental characteristics and disparities in adult asthma in north central Texas urban counties	asthma	urban counties in North Central Texas (Collin, Dallas, Denton, and Tarrant)	Texas counties
Lin et al., 2022 [20]	The associations between residential greenness and allergic diseases in Chinese toddlers: A birth cohort study	asthma, eczema, atopic dermatitis, urticaria, allergic rhinitis, allergic conjunctivitis, food allergy	Guangzhou, China	522 mother-child pairs
Lotfata et al., 2023 [49]	Socioeconomic and environmental determinants of asthma prevalence: a cross-sectional study at the U.S. County level using geographically weighted random forest	asthma	3059 counties in the USA	3059 counties in the USA
Duquesne et al., 2023 [50]	The influence of urban trees and total vegetation on asthma development in children	asthma	island of Montreal, Canada	352,946 children born between 2000–2015
Lovasi et al., 2013 [51]	Urban Tree Canopy and Asthma, Wheeze, Rhinitis, and Allergic Sensitization to Tree Pollen in a New York City Birth Cohort	childhood asthma, wheeze, rhinitis, and allergic sensitization	New York city, USA	549 Dominican or African-American children born in 1998–2006
Lukkarinen et al., 2023 [52]	Early-life environment and the risk of eczema at 2 years-Meta-analyses of six Finnish birth cohorts	eczema	Finland	5085 children were obtained from six Finnish birth cohorts
Maio et al., 2022, 2022 [53]	Urban grey spaces are associated with increased allergy in the general population	allergy	Pisa/Cascina, Italy	2070 subjects (age range 15–84 yrs)
Malamardi et al., 2022 [54]	Time Trends of Greenspaces, Air Pollution, and Asthma Prevalence among Children and Adolescents in India	asthma	29 states and one union territory of India	millions of childen, 0–19
Mansouri et al., 2024 [55]	Residential surrounding greenness and the incidence of childhood asthma: Findings from a population-based cohort in Ontario, Canada	asthma	Ontario, Canada	982,131 children and mother-offspring pares
Markevych et al., 2020 [56]	Residing near allergenic trees can increase risk of allergies later in life: LISA Leipzig study	Asthma and allergic rhinitis, sensitization to aeroallergens and food allergens	Leipzig, Germany	631 children
Nurminen et al., 2021 [57]	Land Cover of Early-Life Environment Modulates the Risk of Type 1 Diabetes	type 1 diabetes	Oulu, Tampere, and Turku, Finnland	10,681 children genotyped for disease-associated HLA-DQ alleles,271 of them developed type 1 diabetes and 384 of them had multiple diabetes-associated islet autoantibodies, born between 1994 and 2013 and till 15 years or clinically diagnosis of type 1 diabetes
Paciência et al., 2021 [58]	Neighbourhood green and blue spaces and allergic sensitization in children: A longitudinal study based on repeated measures from the Generation XXI cohort	allergic sensitization	Nothern Portugal	730 children at the age of 10
Paciência et al., 2022 [59]	Association between Land Use Mix and Respiratory Symptoms and Asthma in Children from the Generation XXI Birth Cohort	asthma, respiratory symptoms	Portu mentropolitan area, Portugal	6260 children
Parmes et al., 2020 [60]	Influence of residential land cover on childhood allergic and respiratory symptoms and diseases: Evidence from 9 European cohorts	asthma, wheezing, allergic rhinitis, eczema	Europe ((Italy, France, Slovenia and Poland), nine cohorts	8063 children, aged 3–14 years (nine cohorts)
Putra et al., 2022 [61]	Caregiver perceptions of neighbourhood green space quality, heavy traffic conditions, and asthma symptoms: Group-based trajectory modelling and multilevel longitudinal analysis of 9,589 Australian children	asthma	Australia	9589 children, 12–15 y.o., observed for 10 years
Rantala et al., 2023 [62]	Green space during pregnancy and the development of asthma up to 27 years of age: The Espoo Cohort Study	asthma	Espoo, Finland	children delivered between 1 January 1984 and 31 March 1990
Cavaleiro Rufo et al., 2021 [63]	The neighbourhood natural environment is associated with asthma in children: A birth cohort study	asthma and allergic diseases	Portu metropolitan area, Portugal	1050 children
Razavi-Termeh, 2021 [64]	Spatial Modeling of Asthma-Prone Areas Using Remote Sensing and Ensemble Machine Learning Algorithms	asthma	Tehran, Iran	872
Squillacioti et al., 2020 [65]	Greenness Availability and Respiratory Health in a Population of Urbanised Children in North-Western Italy	asthma and other health issues (not IMID)	Turin, Italy	187 children, 10–13 y.o.
Stas et al., 2021 [66]	Residential green space types, allergy symptoms and mental health in a cohort of tree pollen allergy patients	allergy	Belgium	157 adults with pollen allergy
Stas et al., 2021 [67]	Exposure to green space and pollen allergy symptom severity: A case-crossover study in Belgium	allergic rhinitis	Belgium	144 allergy patiens
Turunen et al., 2023 [68]	Cross-sectional associations of different types of nature exposure with psychotropic, antihypertensive and asthma medication	asthma and other non-IMIDs	Helsinki, Espoo and Vantaa, Finland	7321
Wang et al., 2022 [69]	Preplanned Studies: Greenness and Asthma in the Middle-Aged and Elderly Population in a Prospective Cohort Study — China, 2011–2018	asthma	125 cities and 28 provincial-level administrative divisions in China	17,574 adults middle aged and elderly population, CHARLS Cohort followed in 2011–2018 (670 new-onset asthma cases)
Wu et al., 2021 [70]	Greenness and eosinophilic asthma: findings from the UK Biobank	eosinophilic asthma	UK	351,717 adults aged 37 to 73 years
Yu et al., 2021 [71]	Associations between trees and grass presence with childhood asthma prevalence using deep learning image segmentation and a novel green view index	asthma	Notheast China	59,754 children aged 2–17 years that did not change their residental address
Zaldo-Aubanell et al., 2022 [72]	Environmental heterogeneity in human health studies. A compositional methodology for Land Use and Land cover data	asthma and other health issues (not IMID)	Spain	whole population of Catalania with asthma and other diagnosis
Zeng et al., 2020 [73]	Greenness surrounding schools is associated with lower risk of asthma in schoolchildren	asthma	Seven Northeast Cities Study, China	59,754 schoolchildren from 94 schools

**Fig. 2:**
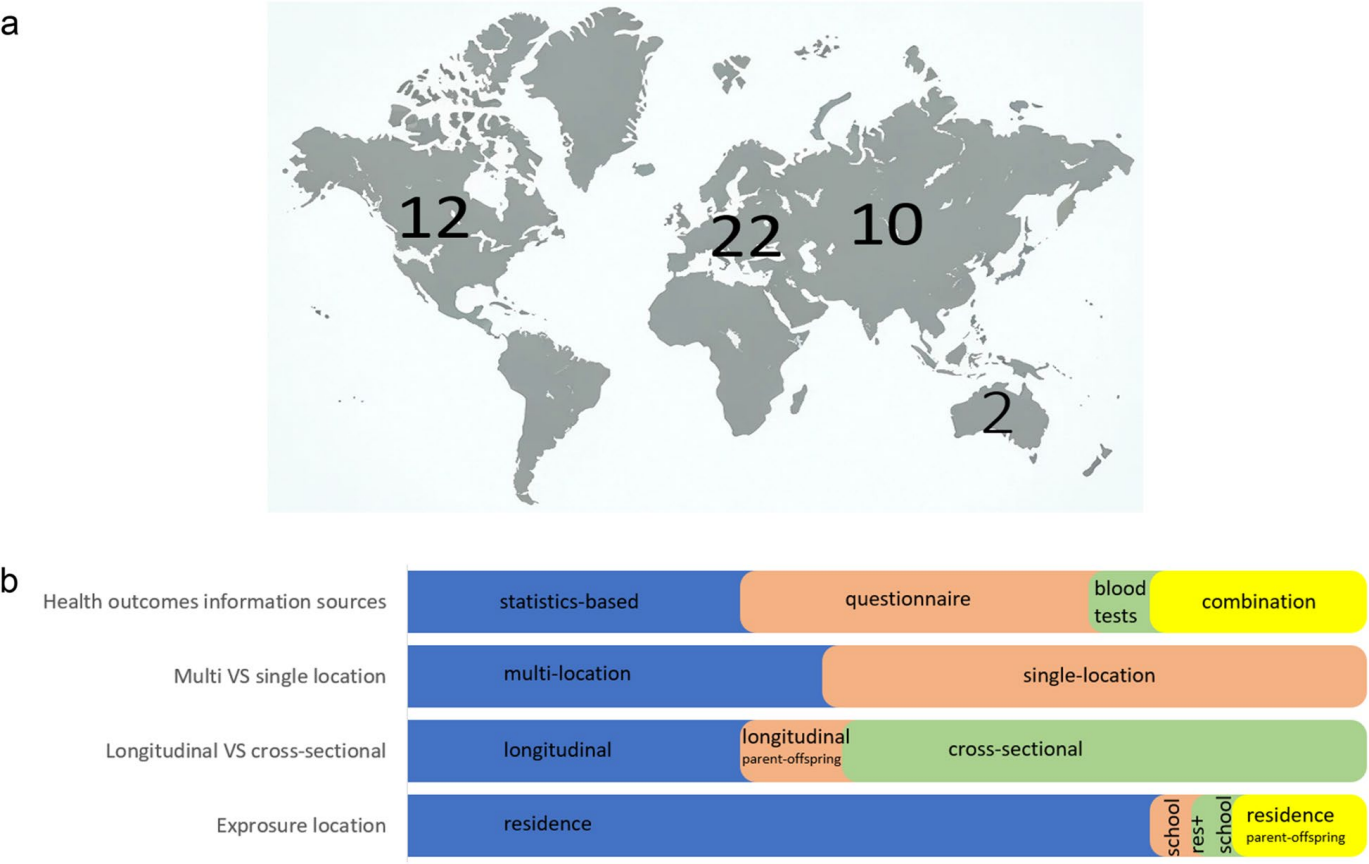
Characteristics of the original research studies included in the Review: (**a**) Geographic distribution of the studies by continent; (**b**) details on the information sources utilized, the count of multi-location versus single-location studies, the breakdown of longitudinal versus cross-sectional studies, and the distribution of studies according to different exposure locations

All the publications were observational, 17 included statistical data about health from National registries and hospitals, 17 were based on self-reported and doctor diagnosed diseases symptoms revealed from questionnaires, three were based on blood testing and the other nine combined blood testing with questionnaire and/or statistic data (Fig. 2).

None of the studies included double-blinded, placebo-controlled intervention trials. Indeed, green spaces represented by trees, shrub and grass is difficult to control in such experiments. We found a publication concerning environmental effects on immune mediated diseases that was organized as placebo-controlled intervention trial. It was dedicated to the testing of influence of diverse microbial community on immune system though, and therefore did not meet the criteria of our search strategy [7, 74].

Among 46 publications, 17 represented longitudinal studies, 24 – cross-sectional studies, and five longitudinal studies aimed to describe how parent’s exposure to nature affect their offspring’s IMIDs. Concerning geographic scale, 21 studies were conducted on multiple and 25 - on one geographic location (city, megacity, metropolitan area or region). The number sample ranged from 144 to 982,131 people, with varying demographic characteristics: children, toddlers, parents-offspring pairs, adults, youth, all ages. Additionally, some studies examined disease frequencies in large groups (several million people) residing in specific regions. The research was conducted in Europe (Belgium, Germany, Italy, Austria, Finland, Sweden, Spain, Norway, Portugal, France, Slovenia, Poland, the UK) – 22 papers, North America (Canada, the USA) – 12 papers, Asia (China, Taiwan, India, Iran) – 10 papers, and Australia and Oceania (Australia, New Zealand) – two papers.

### Participants of the reviewed studies

32 of 46 reviewed studies involved children as participants. Of these, four were centered on infants and toddlers, monitoring their health status from birth. Another five studies examined the relationship between parents and offspring, focusing on the prenatal period and early life exposure to green spaces, among other topics. The majority of the studies, numbering 23, targeted daycare or school-aged children. However, only two of these specifically considered the school’s address, while another two assessed both the school and home addresses for estimating exposure to green spaces. The authors assumed that school children spend a lot of time at schools, and therefore school green space exposure plays an important role for their health. Indeed, it was demonstrated that greenness surrounding schools is associated with lower risk of asthma [73]. In one of the publications where authors combined information about school and home green space coverage, different effects of school versus residential greenness were found [32].The remaining studies used the residential address to estimate green space exposure. A minority of the studies did not specifically focus on children, with seven studies involving only adults and another seven including participants regardless of age.

### Metrics used to describe human exposure to vegetation

In the publications reviewed, different terms have been used to describe the availability/provision of green environments and to estimate the exposure of humans to the vegetation. Particularly, green space was characterized using several completely or mostly overlapping terms, most notably green space, green area, greenness, vegetation cover, tree canopy, naturalness. All of them described the area covered by plants, with the exception of tree canopy that covers just trees and other woody plants.

**Fig. 3:**
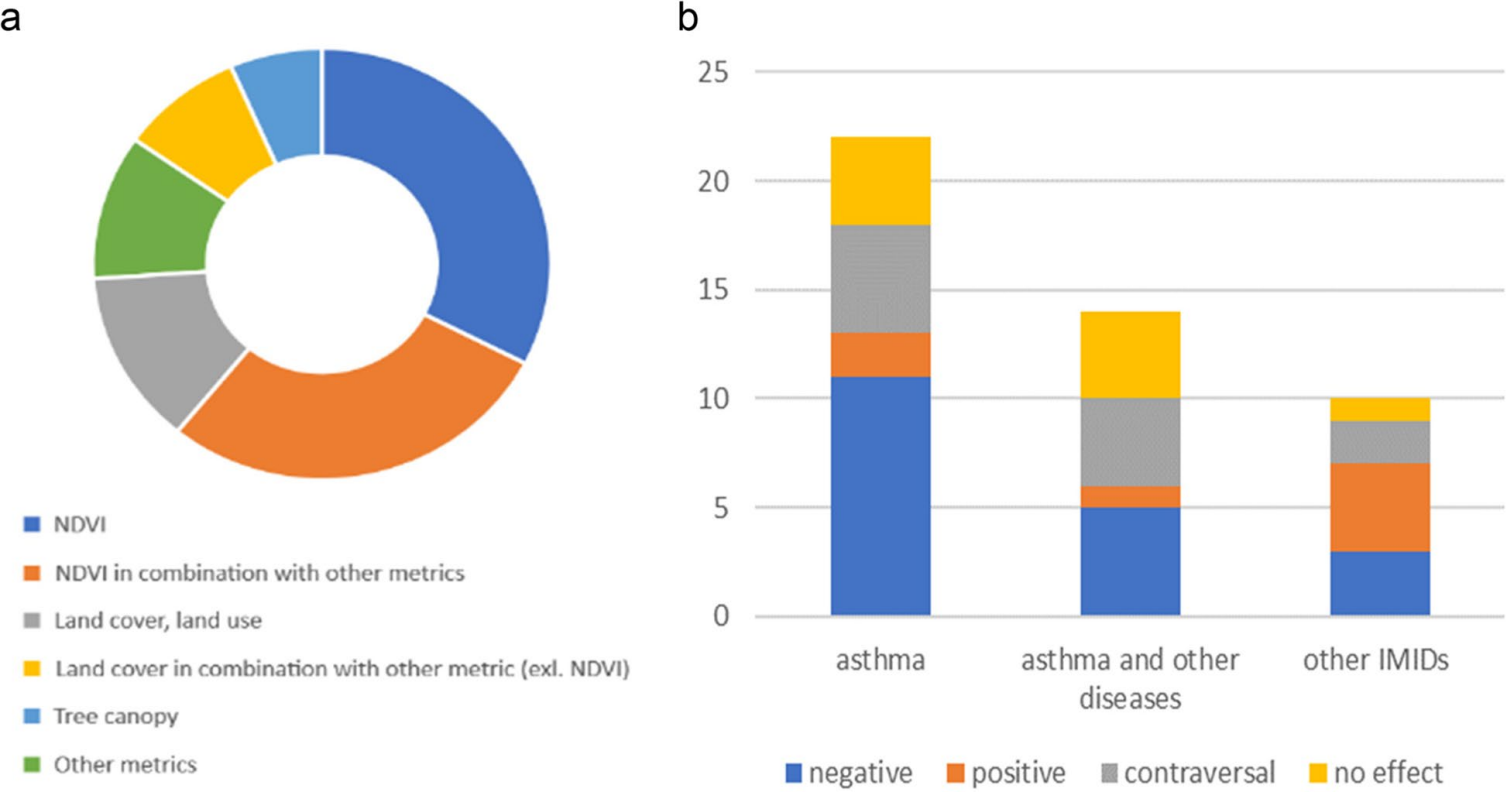
The green space metrics employed in the studies reviewed (**a**) and the identified associations between green spaces and risks of IMIDs (**b**)

To measure green area, normalized difference vegetation index (NDVI) was used more often than other methods; in 15 publications NDVI was the only metrics used to estimate green space coverage (greenness), in 13 other publications NDVI was used in combination with data about species diversity, tree canopy index, land cover data, and/or other green space metrics proposed by the authors (e.g. Google street view index) (Fig. 3a). In total, 61% of the publications analyzed in the current review included NDVI measurements of different scales and precisions. Land cover (or land use) type was the second popular metrics among publications analyzed in this review. 10 of 46 studies used land cover information (solely or in combination with other metrics) to calculate the green space exposure level, three of them used the information from CORINE (*coordination of information on the environment*) database, and others consumed information from National databases and registers.

To describe exposure to green spaces, most studies employed the concept of a “buffer zone,” which refers to a designated area around an individual’s home or school. This method quantifies green space exposure by measuring the size of the green area within a predetermined distance from a central location, usually where the participant lives or spends a considerable amount of time. Other, less commonly used metrics for evaluating exposure to green spaces included the frequency of visits to green spaces, the proximity to green spaces, and the configuration of green space patches. The circles with a radius of 7.5, 50, 100, 200, 250, 300, 500, 1000, 1500, 5000 and 5000 m with the center corresponding to the study participant’s residence or school address were assumed to be accurate in the studies analyzed in the present review. In some cases, authors calculated relationships between green spaces exposure and health outcomes and compared them for several radiuses chosen. Among publications analyzed in our review, 11 used more than one radius to characterize the “influencing” zone, and at least three of them demonstrated different and even controversial results with different radiuses [20, 32, 63].

### IMIDs analyzed in the original research studies and the observed effects

The most of the publications analyzed participants with asthma only (22) or with asthma and other health outcomes such as allergic rhinitis, allergies, eczema, atopic dermatitis, urticaria, allergic conjunctivitis, hey fever, wheezing, atopic sensitization (14) (Fig. 3b). The other 10 publications concerned childhood acute lymphoblastic leukemia, eczema, allergy, allergic sensitization, allergic rhinitis and type 1 diabetes but not asthma.

In 20 publications, modelling and statistical processing of data in order to reveal associations between green space coverage and IMID risks demonstrated absence of correlation, inconsistent results or contrasting results at different radii. 19 studies demonstrated a decreased IMID risk among study participants exposed to green spaces. Finally, seven publications demonstrated an increased IMID risk for participants exposed to higher green space coverage.

We attempted to identify the factors influencing the consistency of study results. Potential factors included cohort size, participant age, exposure location, green space metrics, IMID assessment type, and study design (longitudinal vs. cross-sectional). However, the dataset size of available literature was insufficient for statistical analysis. A simplified quality analysis, calculating the percentage of consistent results for each factor, also failed to identify a single significant contributor to result consistency. Nevertheless, two studies comparing NDVI with other green space metrics found that vegetation diversity and land cover, rather than NDVI, correlated with IMID risk, suggesting that excluding NDVI may improve result consistency [39, 40]. In contrast, no longitudinal studies found the opposite, i.e., that NDVI revealed a reduced disease risk while more specific green space metrics did not. In addition, using finer spatial resolutions [75] and assessing green space accessibility and use, and adopting longitudinal or experimental designs to establish causality were encouraged in the reviewed literature and an earlier study [5].

## Discussion

### Inconsistency of results and conclusions of the original research studies

The results of publications from 2020 to 2024 demonstrate controversial associations between greenness and IMIDs. These discrepancies reflect how sensitive age range varies from disease to disease, and even depends on environmental conditions [68].

We observed the publications included in the present review to have the following features. First, large cross-sectional studies involving thousands of people tended to use relatively simple methodology to estimate green spaces coverage and exposure, i.e., only NDVI with one buffer zone was used together with residence address of the participant, instead of birth address. Second, studies with smaller sample sizes tended to go deeper in details, improve the way of green spaces measurements, including vegetation quality assessment, and often conduct longitudinal studies. Obviously, the optimum would be a combination of large number of study participants and the deep analysis of green environment, including the separation of vegetation types and use of several buffer zones as was done by Nurminen et al. [57].

Since inconsistences between studies at least partially follow from the differences in study design described above, the data are not comprehensive enough to make final conclusions of associations between urban green spaces – their area and structure –and IMID risks and to publish exact recommendations for urban planning and human behavior [13, 21–23]. These uncertainties, however, do not overwrite the general recommendation that daily exposure to microbiologically diverse green space promotes health and wellbeing particularly among children and vulnerable people [68]. The unified methodology for revealing the effects of green spaces on immune health is urgently required and it would be pivotal to form an international evaluation body to set up standard criteria for the forthcoming studies. Cardinali et al., recognizing the critical need for a standardized methodology, proposed a provisional solution. Specifically, they developed the “Preferred Reporting Items in Greenspace Health Research” for studies investigating the relationships between green spaces and health outcomes76 [76]. According to the authors, the proposed guidelines aim to facilitate high-quality assessments and harmonize research in this field, while also accommodating the diversity of study designs.

Both original researches and reviews, including meta-analyses, frequently highlight that the varied impacts of green spaces on the risk of IMIDs stem from multiple underlying causal relationships or mechanisms, rather than a singular cause-and-effect pathway. These mechanisms can produce contrasting outcomes. For instance, plants, especially trees, can act as sources of pollen, which is known to worsen immune diseases such as asthma and allergic rhinitis. Conversely, green vegetation can also absorb pollutants from the air, thus alleviating the impact of these pollutants on the aggravation of IMIDs [27, 44, 66]. The aforementioned mechanisms and their contradictory effects will be elaborated upon further below.

### Potential reasons for inconsistent results

Based on our analysis of the original research studies, we propose two main factors that may explain the inconsistencies in the results obtained by different authors over time. These are: (1) huge differences in study methodologies and design, and (2) the presence of multiple, often contradictory, mechanisms through which vegetated surfaces affect human immune health. The dominance of one mechanism over another during the research period, for a specific participant group, can greatly influence the study’s outcomes. Below, we discuss both factors in greater detail.

## Differences in study design and methodology

### Methodologies used for analyzing green space exposure

The methodologies for analyzing green space exposure can be categorized into two main approaches. The first focuses solely on estimating green space coverage through various metrics. This estimation can range from complex methods, such as utilizing Google Street Views or LiDAR (multispectral satellite imagery with the assistance of light detection and ranging) data, to more simplified approaches using NDVI. These methods primarily measure the extent of greenness. The second, more comprehensive methodology encompasses not only the metrics of green space but also factors related to green space exposure. This includes the frequency of visits to green areas, size of green area the participant is exposed to, the duration of residence in a particular environment, time spent at educational institutions, or the influence of different seasons. This holistic approach aims to capture both the quantitative aspect of green spaces and the qualitative experience of individuals with these environments.

Both original researches and reviews, including meta-analyses, frequently highlight that the varied impacts of green spaces on the risk of IMIDs stem from multiple underlying causal relationships or mechanisms, rather than a singular cause-and-effect pathway. These mechanisms can produce contrasting outcomes. For instance, plants, especially trees, can act as sources of pollen, which is known to worsen immune diseases such as asthma and allergic rhinitis. Conversely, green vegetation can also absorb pollutants from the air, thus alleviating the impact of these pollutants on the aggravation of IMIDs [27, 44, 66]. The aforementioned mechanisms and their contradictory effects will be elaborated upon further below.

### Green space metrics

The findings in the most recent publications, ranging from 2020 to 2024, corroborate the outcomes of earlier reviews and meta-analyses [24–28, 77–83]. Specifically, the most frequently utilized metrics for green space are NDVI, land use cover, and tree canopy coverage. Indeed, NDVI is calculated on the basis of satellite data and therefore is available on every geographic location on the globe. While using NDVI, it is commonly assumed that the results obtained in different studies are comparable. However, NDVI seems to suffer from several limitations that may partly result in the inconsistent findings of greenspace exposure and health. The main limitations are low resolution, impossibility to distinguish between different vegetated surfaces and vegetation types, like coniferous versus mixed versus deciduous forests, and shrubs versus trees versus non-woody plants; and no information about species diversity [75]. Another limitation may be that the scale is too large in order to identify green space provision for individual households in their own environment [71]. Notably, in studies covered by the current review, the association between several IMIDs and green spaces varied with vegetation type (agricultural areas versus forest, broadleaved versus coniferous forests) as well as different tree species (allergenic, non-allergenic), particularly for the incidence of allergic rhinitis and asthma but also for type 1 diabetes. Yu with co-authors (2020) described one more limitation of NDVI: it assesses the so called bird’s-eye overhead view of greenness that does not correspond to the eye-level of greenness, while particularly eye-level greenness might influence human behavior and thus health. Apparently, the same limitation of NDVI is related to its inability to evaluate the possibility for close contacts, such as crawling and touching that both have been shown to effectively change commensal microbiota [84–87] and immune response [7, 74, 88]. For further epidemiological investigations, the authors suggested the novel index that combines street view and uses artificial intelligence to extract green spaces metrics [71]. Another attempt to overcome the limitations of NDVI was the use of the data of the vegetation derived from multispectral satellite imagery with the assistance of light detection and ranging (LiDAR) information.

### Green space exposure characteristics

The correct determination of the locations where individuals are exposed to green spaces seem to play the most important role for the accuracy of the results. The determination itself and collection of data for many individuals that can change residence addresses, travel or make daily movements from home to work or school is costly to do comprehensively. Many researchers have simplified their study design by assuming that the immediate neighborhood of the home address is the primary environmental area determining the risk of IMIDs although other environments were study subjects have interactions with green spaces may be equally or more important [75].

The correct determination of the locations where individuals are exposed to green spaces seem to play the most important role for the accuracy of the results. The determination itself and collection of data for many individuals that can change residence addresses, travel or make daily movements from home to work or school is costly to do comprehensively. Many researchers have simplified their study design by assuming that the immediate neighborhood of the home address is the primary environmental area determining the risk of IMIDs although other environments were study subjects have interactions with green spaces may be equally or more important [75].

The size of the zone that is significant for the human health is another important question that researches have to answer while planning the study. Most likely, the size of the “influencing” zone is dependent on the movement activity and habits of the study participant, landscape and wind flow characteristics, temperature and other factors. For every study, a reasonable and everyone’s acceptable radius should be chosen and substantiated in order to obtain consistent and comparable results, since using different selection methods for radiuses may lead to different conclusions with the same data set. Authors from Singapore demonstrated that the results on asthma prevalence and its dependence on green spaces may differ when different metrics as well as different types of buffer area or different spatial scales are chosen by the researchers [71]. American researchers confirmed the importance of spatial resolution for the findings concerning relationships between urban green areas and human health [89]. There are studies that underline the importance of humans’ actual exposure to green spaces (e.g. the number and duration of visits) above residential green space, independent on its size [68]. In many studies, although the health benefits of green spaces may extend across considerable distances (for example, when residents travel from distant areas to access green spaces), practical limitations often prevent the use of large buffer zones. Conversely, in study areas with different characteristics, the opposite situation may arise. As researchers aim for strong causal relationships in their findings, this may require using the smallest feasible buffer zone to exclude health effects from nearby areas (such as parks, playgrounds, or rivers).

A noteworthy characteristic of exposure to green spaces, observed in several studies reviewed, pertains to the seasonality of the analysis [50]. For instance, exposure during the third trimester of pregnancy in the warmer seasons of spring or summer has been shown to differentially impact the incidence of asthma in offspring [62]. Another study revealed that exposure to green spaces in early childhood during spring increases the likelihood of developing allergic rhinitis in adulthood, while exposure during summer has a protective effect. This disparity is proposed to stem from the simultaneous yet opposing influences of pollen exposure in spring and the mitigation of air pollution by vegetation in summer [44].

In can be concluded that the methodology for estimating green spaces requires refinement and advocate for a standardized approach, as the results are currently method-dependent and not comparable. Additionally, they highlight that a direct correlation between green space coverage and immune-mediated diseases cannot be established due to the significant influence of other factors. These include the frequency of visits to green spaces, the quality of these areas, the allergenic potential of the trees, and seasonal variations—such as the increased leaf surface area in the summer, which can absorb atmospheric pollutants and reduce their adverse effects on individuals with asthma, among other factors [28, 29, 44, 56, 61, 67, 68].

### Methodologies used for analyzing health outcomes

The studies we analyzed varied significantly in terms of health outcomes. As with previous reviews, asthma was identified as the most frequently analyzed IMID [24]. However, even in the case of asthma, the methods used to characterize patients varied, ranging from self-reported symptoms to number of emergency room visits or hospitalization.

In the reviewed studies, a diverse array of methodologies was employed to investigate disease incidence. These included the analysis of hospital statistical data, self-reported questionnaires on doctor-diagnosed conditions, and assessments completed by healthcare professionals. Furthermore, several studies utilized or exclusively depended on a range of diagnostic tests, such as blood tests, evaluations of lung volume, and tests for allergic reactions.

The methodologies for selecting study participants were notably diverse across the reviewed publications. The size of the study groups ranged from a mere handful to several million participants, encompassing a broad spectrum of age groups. This included parent-offspring duos, early life children, children, adolescents, adults, the elderly, and groups where age was not a discriminating factor. Furthermore, strategies for participant selection also varied widely. In some instances, researchers examined the entire population of a specific area. In others, individuals were chosen based on their genetic susceptibility to IMIDs. The review also encompassed findings from particular prospective population cohorts, whose attributes were meticulously observed over prolonged periods.

Prenatal period and early years of life are reported to be important for maturation of gut and skin microbiota as well as for immune system development [20, 57]. Moreover, in early life children typically live at home or around home and therefore it is particularly relevant to measure green space exposure using residential address. A substantial portion of the studies specifically targets school-aged children. The reason for such an age focus might be the relative simpleness of data collection for school children, with the help of school personnel and health care system or parents, and the first manifestations of IMIDs that already occur at the age under 3 years and later in childhood.

Interestingly, only one of the reviewed studies in the current review dealt with cases belonging to a genetically determined IMID risk group, and the others dealt with study participants of different ages but with no relevance to IMID risk. In the work of Nurminen and co-authors, newborn children confirmed to carry a genetic risk for type 1 diabetes based on HLA-DQ typing, and who lived at least one year in same address were included and followed-up for 15 years or for the onset of T1D [57]. Such an approach seems to be promising to obtain precise associations between the IMID risk and exposure to nature. A drawback is that the results can be obtained only in a decade after the research starts.

### Potential mechanisms behind associations between green spaces on IMID risk

**Fig. 4:**
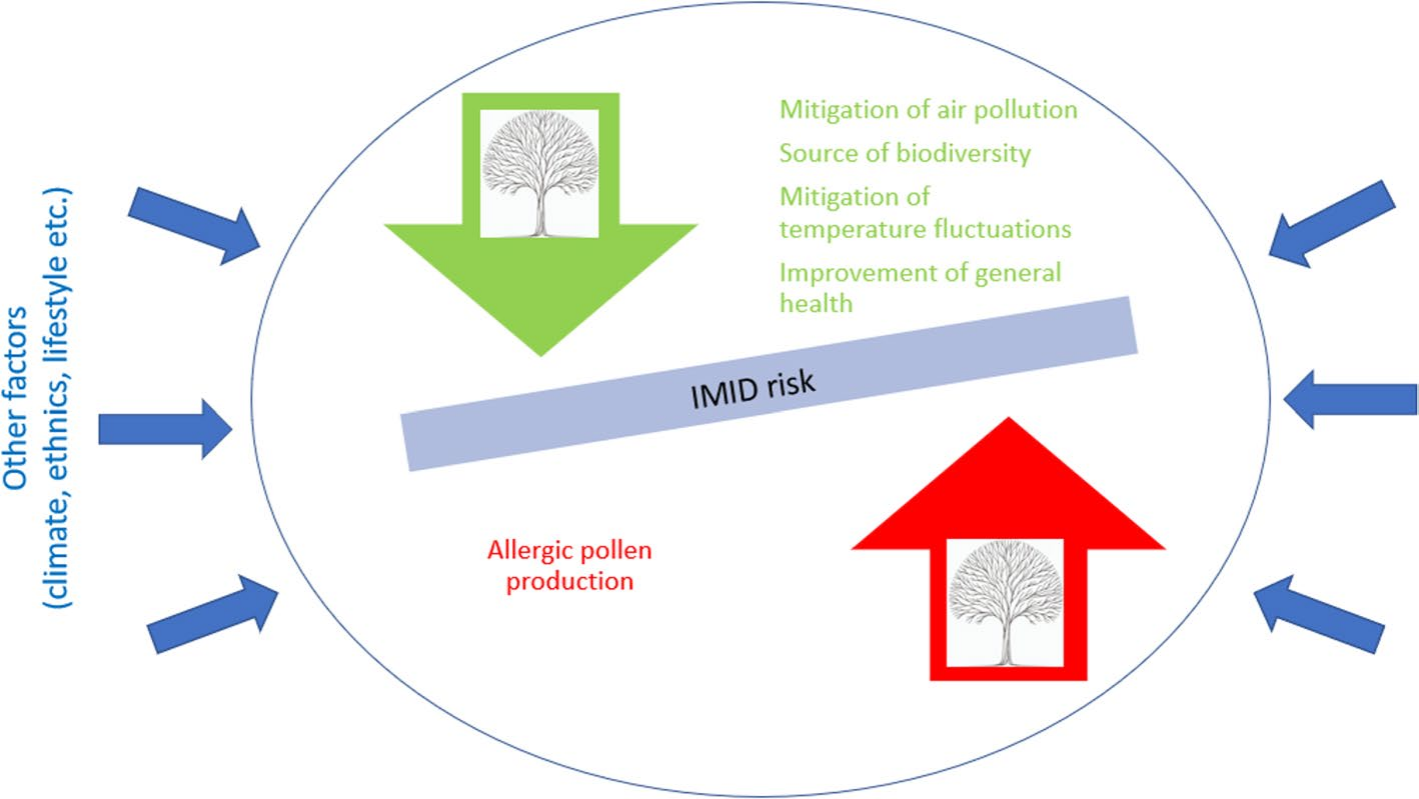
Potential mechanisms underlying the impact of green spaces on the IMIDs risk

Since most comparative publications propose a mechanism how green space affects the IMID risk, it is worthwhile to list the mechanisms, albeit we do not intend to search for a consensus about underlying mechanisms. The mechanisms proposed cover the effects of exposure to microbial diversity, plant pollen and other plant produced allergens; positive influence of microbial diversity (Fig. 4) [7, 60, 61, 76, 87, 90–105]. Moreover, green spaces are shown to mitigate air pollution and partially also noise, visiting nature area shown to improve of mental and physical health. Vegetation indeed is the sole source of pollen and a main source of molds, aerosols and volatile organic compounds that play a role in allergen exposure and might cause IMID manifestation. It should be noted though that not every pollen possesses allergenic characteristics, thus the effect of green spaces will be rather determined by species composition but not vegetation cover [31, 60, 90–92]. The hypothesis about connection between IMIDs and microbial diversity is known under “old friends”, “hygiene” and “biodiversity” titles and was proposed by Strachan (1989), Rock et al. (2003) and von Hertzen et al. (2011) [93–95]. It is often assumed that the diversity of skin and gut microbiota is inversely correlated with IMID prevalence since diverse microbiota encourages maturation of the immune system [96]. Recent intervention trials demonstrate that the hypothesis has sound basis, i.e., that urban environment reduces transfer of soil microbiota onto skin, that skin microbiota of urbanites becomes more diverse in soil exposure, and that immune modulation is enhanced among those who host diverse microbiota [7, 87, 97, 98]. In addition, skin and gut microbiota have been observed to be influenced by soil and air microbiota that in turn are shaped by surrounding trees [86, 99, 100]. Finally, since heat islands may increase IMID risk or worsen their symptoms, and since vegetation cuts weather extremes [92, 101, 102], green space is likely to provide ecosystem services in multiple ways that together shape the IMID risk and manifestations.

One of the factors behind the discrepancy of associations between green space and IMID risk may be the ability to filtrate ambient air and facilitate the sedimentation of atmospheric aerosols [100, 103]. NO_X_, O_3_, polyaromatic hydrocarbons, particular matter of 5 and 2.5 μm were demonstrated to mediate IMID risk while plants were proposed to mitigate this effect. Obviously, the potential mitigation varies depending on leaf area, stomata structure and other individual characteristics of plant community [92, 104, 105]. In addition to factors mentioned above, “heat island” effect of the cities, i.e., high temperatures and windless weather, have been demonstrated to increase IMID risks [92, 101, 102]. To summarize, the weight of each mitigation mechanism should be modelled in order to fully understand the role of green spaces in IMID risk. Attention should be paid to distinguishing between correlation and co-occurrence between the green space characteristics and IMID prevalence.

## Concluding remarks

The interest of the scientists and urban managers to the IMID risk associations with green space coverage in cities remains high, and variously designed studies are conducted in order to reveal the presence and mechanisms of those associations. Unfortunately, the hypothesis that any green at any age is equal seems an oversimplification in the context of human health. Therefore, the inconsistent results of the reviewed studies that had different study designs, methodologies, and disease assessment criteria are not surprising. As known from clinical studies, IMID risk varies with age, lifestyle, and environmental influences. Furthermore, conflicting mechanisms by which vegetation and various vegetated environments impact human health could also contribute to the variability in findings across different research studies.

Of the 46 studies analyzed in this review, 26 provided clear conclusions about the effect of vegetation on IMID risk. Of these, 19 demonstrated a protective effect, which is promising and encourages further investigation. However, the development of standardized methodologies is crucial to enable meaningful comparisons and meta-analyses. Several recommendations from the authors of the original research articles included in this review should be considered for future study designs. The future studies should include larger participant cohorts (ranging from several thousand to millions) and employ more accurate methods for estimating exposure to green spaces than the current reliance on NDVI. For this, manual registration, sampling and artificial intelligence to process street view images are encouraged. National registries and geolocation might provide larger and more precise data sets in the future. Standardizing exposure characteristics, including location, buffer zone size, and exposure timing, is crucial for obtaining consistent, comparable results. Finer spatial resolutions and varied exposure assessment methods should be adopted. The future studies should incorporate assessments of environmental microbial diversity. In addition, incorporating placebo-controlled trials using longitudinal approaches is recommended. Studies should prioritize birth or early-life addresses in parallel with current addresses, as early exposure may be more significant in certain diseases. Moreover, the impact of maternal exposure during pregnancy on offspring IMID risk requires further research.

Finally, since there evidently is a huge need to understand the associations between the living environment and human health, we propose an international standardization body to set up and manage study methodologies, designs and outcomes and to guide the future attempts in the field of land cover and green space oriented human and planetary health research for understanding of any potential causality between the exposure and the outcome.

## Supplementary Information

Supplementary Material 1.

## Data Availability

No datasets were generated or analysed during the current study.
